# Harnessing adipogenesis to prevent obesity

**DOI:** 10.1080/21623945.2019.1583037

**Published:** 2019-03-08

**Authors:** Nida Haider, Louise Larose

**Affiliations:** Division of Experimental Medicine, Department of Medicine, McGill University and The Research Institute of McGill University Health Centre, Montreal, Quebec, Canada

**Keywords:** Adipocyte precursor cells, adipocyte, adipogenesis, white adipose tissue, obesity, Aregs, PDGFRα, miRNA, lncRNA

## Abstract

Obesity and associated metabolic complications, including diabetes, cardiovascular and hepatic diseases, and certain types of cancers, create a major socioeconomic burden. Obesity is characterized by excessive expansion of white adipose tissue resulting from increased adipocyte size, and enhanced adipocyte precursor cells proliferation and differentiation into mature adipocytes, a process well-defined as adipogenesis. Efforts to develop therapeutically potent strategies to circumvent obesity are impacted by our limited understanding of molecular mechanisms regulating adipogenesis. In this review, we discuss recently discovered molecular mechanisms restraining adipogenesis. In this perspective, the discoveries of white adipose tissue endogenous adipogenesis-regulatory cells (Aregs) that negatively regulate adipocyte differentiation, platelet-derived growth factor receptor isoform α (PDGFRα) activation and downstream signaling that hinder adipocyte precursors differentiation, and a group of obesity-associated non-coding RNAs (ncRNAs) that regulate adipogenesis open up promising therapeutic avenues to prevent and/or treat obesity.

## Introduction

Under physiological conditions, human body weight is governed by a balance between energy intake and energy expenditure. In this regard, excessive calorie input without proper adjustment of calorie used through daily activities or resulting from adverse hormonal metabolic milieu contributes to the development of obesity [1]. In recent years, obesity has become an extremely prevalent phenomenon which is still poorly understood from an etiologic-mechanistic perspective. Indeed, worldwide in 2016, an estimated 1.9 billion of the adults, 18 years and older, were overweight and with over 650 million of these were obese [2] and effective pharmacological approaches available to prevent the development of obesity are very few. Given that obesity is a major risk of life-threatening diseases such as cardiovascular diseases, type 2 diabetes, non-alcoholic fatty liver disease (NAFLD) and cancer [2–4], obesity is now amongst the serious health issues.

**Figure 1.**
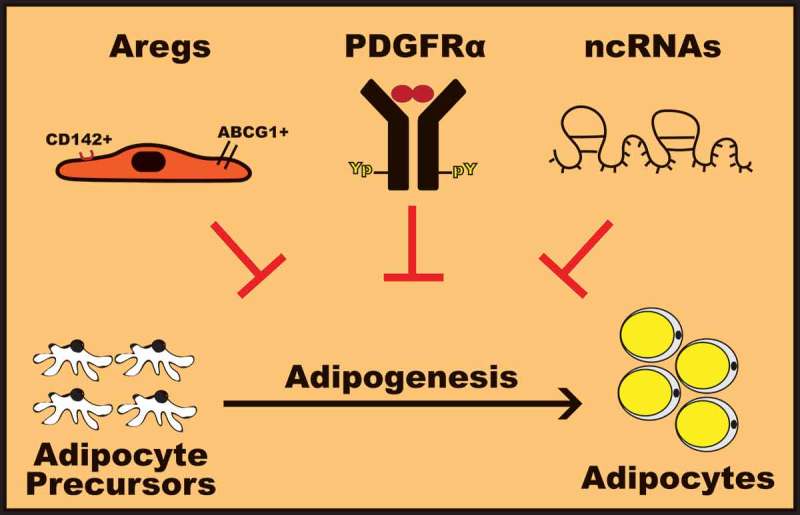
Molecular mechanisms to harness adipogenesis. Differentiation of adipocyte precursor cells into adipocytes could be hindered by adipogenesis-regulatory cells (Aregs), PDGFRα activation and signaling, and non-coding RNAs (miRNAs and lncRNAs). The figure is created using Adobe Illustrator CC.

Obesity is characterized by an excessive accumulation of white adipose tissue (WAT). WAT, mainly composed of adipocytes that store lipid, plays an important role in the control of whole-body energy homeostasis [1]. During normal development and abnormal expansion associated with obesity, WAT remodeling involves hypertrophy (increase in size) and adipocyte hyperplasia (increase in number) [5,6]. Adipocyte hyperplasia, defined as adipogenesis, is to a certain extent linked to the healthy expansion of WAT through proliferation and differentiation of adipocyte precursor cells (APCs) present in WAT stromal-vascular fraction (SVF) [6]. In contrast, hypertrophic WAT expansion due to enlargement of existing adipocytes, along with macrophage infiltration and fibrosis, contributes to WAT dysfunction associated with insulin resistance and metabolic syndrome [6,7].

The process of adipogenesis is already well reviewed in various landmark publications [8–10]. Briefly, adipogenesis involves the regulation of a complex network of various transcription factors, which among others include the master regulators peroxisome proliferator-activated receptor gamma (PPARγ) and the family of CCAAT enhancer binding proteins (C/EBPs) [8–10]. Indeed, PPARγ along with C/EBPα function to control the transcription of various target genes that establish the adipocyte signature. However, through the years, additional players either potentiating or inhibiting differentiation of adipocyte precursor cells into adipocytes have been identified. In this review, we focus on the recent discoveries of novel molecular mechanisms limiting adipogenesis and their potential implications in preventing or treating obesity.

## Adipogenesis-regulatory cells (Aregs)

Recently, Schwalie et al. uncovered a novel mechanism regulating *de novo* adipogenic capacity of WAT [11]. Using a single-cell transcriptomics approach, this study revealed the existence of a distinct endogenous WAT SVF cell population displaying a low propensity to differentiate into adipocytes. Interestingly, this subpopulation of SVF cells, characterized by high expression of the cell surface proteins CD142 and the ATP-binding cassette sub-family G member 1 (ABCG1), negatively regulates *ex vivo* mouse and human APCs differentiation in a paracrine manner. Furthermore, the anti-adipogenic function of this SVF cell population is demonstrated *in vivo* by following high-fat diet-induced adipogenesis in mice implanted with matrigel embedded total or CD142^−^ABCG1^−^ SVF cells. Interestingly, matrigel pads containing CD142^−^ABCG1^−^ SVF cells displayed a significantly higher number of mature adipocytes than total SVF cells, further supporting that the CD142^+^ ABCG1^+^ cells prevent adipogenesis *in vivo*. Considering their functional similarity with the T regulatory cells (Tregs) in preventing autoimmunity [12–14], this subpopulation of SVF cells is defined as adipogenesis-regulatory cells (Aregs).

Interestingly, SVF from visceral WAT (vWAT) displays a significantly higher proportion of Aregs as compared to SVF isolated from subcutaneous WAT (scWAT) in humans [11]. It is therefore proposed that the difference in the intrinsic potential of specific WAT depots toward adipogenesis could be attributed to variations in the number of resident Aregs. Following this hypothesis, the higher number of Aregs in visceral compared to subcutaneous fat would suggest that the visceral depot should be more resistant to hyperplasic expansion. However, lineage tracing studies using the AdipoChaser mice, a model tracking adipogenesis *in vivo*, demonstrated higher adipogenesis in epididymal WAT (eWAT) than scWAT upon high-fat diet or cold exposure challenges [15]. The robust hyperplasic expansion of eWAT contradicts the presence of a significantly higher number of Aregs in visceral as compared to the subcutaneous fat depot in humans, weakening the hypothesis that the size of the Aregs population determines the rate of adipogenesis. On the other hand, we and others have reported that in mice, SVF from scWAT displays increased differentiation potential into adipocytes *in vitro* as compared to eWAT [16–19]. Similarly, human adipose stem cells (ASCs) isolated from scWAT have a higher adipogenic potential than vWAT ASCs [16,19], supporting that lower number of Aregs in subcutaneous fat depots may contribute to higher adipogenesis potential. Overall, the contradiction between the number of Aregs cells in visceral and subcutaneous fat depots and their respective adipogenic capacity could be attributed to yet unidentified pro- and anti-adipogenic factors between mice and humans. In addition, higher complexity between *in vitro* and *in vivo* adipogenesis could also lead to different outcomes, thus arising contradictory findings between the correlation of the number of Aregs in various WAT depots with their adipogenic capacity. Nevertheless, the recently discovered existence of the Aregs in various WAT depots potentially provides a novel avenue of investigation to design potential therapies to prevent obesity.

## PDGFRα activation and signaling

Long-term overfeeding induces WAT APCs proliferation and differentiation into mature adipocytes, thus contributing to enhance hyperplasic expansion of WAT leading to obesity [6]. Interestingly, while mature adipocytes lack the α isoform of the platelet-derived growth factor receptor tyrosine kinase (PDGFRα), WAT APCs express PDGFRα [20] and increased number of PDGFRα-positive APCs contributes to the expansion of WAT upon high-fat diet [21]. On the other hand, activation of PDGFRα signaling in APCs blocks differentiation into adipocytes *in vitro* and leads to WAT fibrosis in adult mice due to the conversion of APCs into the extracellular matrix (ECM)-producing fibroblasts rather than adipocytes [22]. Therefore, activation of PDGFRα signaling dictates the balance between adipogenic and non-adipogenic precursor cell populations. Indeed, mice harboring PGDFRα-activating mutations display accumulation of fibroblasts-like stromal cell population associated with WAT fibrosis and reduced embryonic WAT depots [23]. In this perspective, we recently reported that decreased adiposity in mice lacking the Src homology (SH) adaptor protein Nck1 correlates with ECM accumulation in WAT as well as impaired adipogenesis associated with enhanced PDGFRα activation and signaling [18]. Therefore, targeting PDGFRα activation and signaling in APCs may be an interesting avenue to oppose increased adipocyte hyperplasia underlying excessive WAT expansion leading to obesity.

## Non-coding RNAs (ncRNAs)

Evidence of ncRNAs was reported in the early 1980s with the identification of small nuclear RNAs involved in excision of introns. As a result, ncRNAs were considered to be exclusive building blocks of spliceosomes. However, in the early 2000s, the discovery of micro RNAs inducing translation inhibition advanced the field of ncRNAs [24–26]. Important progress in deep sequencing technology has led to the identification of additional members of ncRNA, especially the long non-coding RNAs that emerged as important regulators of cell- and tissue-specific post-transcriptional genes expression. Micro RNAs and long non-coding RNAs involvement in the regulation of adipogenesis and WAT biology is further discussed below.

## Small non-coding micro RNAs (miRNAs)

Small ncRNA miRNAs, which are about 20–25 nucleotides, bind to specific target mRNAs to promote their degradation and/or prevent their translation [27,28]. MiRNAs are detected in all living organisms and actively participate in most normal biological processes, including development, differentiation, and metabolism, but their aberrant expression could result in the development of specific pathologies [29,30]. The mammalian genome is predicted to encode more than 3000 conserved miRNAs [31], among them, several have been investigated in the context of obesity. In fact, an increasing number of genetic and epigenetic studies focusing on obesity revealed miRNAs as potent regulators of post-transcriptional expression of specific genes that are critical in the process of adipogenesis. In this regard, lists of miRNAs that enhance or inhibit adipocyte differentiation in murine preadipocytes or human APCs are reported in recent reviews [32–34]. Briefly, several miRNAs (miR-21, miR-29b, miR-144-3p, miR-148a, miR-210, miR-205-5p) enhance adipogenesis by interfering with the expression of molecular components involved in pathways that counteract adipogenesis (TGF-β, TNF-α, Wnt, corepressors of C/EBPα) [35–40]. Conversely, an important group of miRNAs that inhibits adipocyte differentiation (miR-27a and b, miR-31, miR-128-3p, miR-130a and b, miR-146a-5p, miR-155, miR-540) directly targets master regulators of adipogenic differentiation, such as C/EBPs and PPARγ [41–50]. In accord, these miRNAs are either downregulated during adipocyte differentiation or induced in conditions associated with inhibition of differentiation.

In contrast to the recognized role of miRNAs in regulating adipogenic differentiation, their significance in mature WAT biology still remains elusive. In this perspective, Koh et al. recently reported that in scWAT, lower levels of miR-30a correlate with insulin resistance in diet-induced obese mice and humans [51]. In addition, overexpression of miR-30a in scWAT improves insulin sensitivity and energy expenditure, along with reducing ectopic fat deposition in the liver and inflammation within WAT depots in obese mice. Importantly, miR-30a exerted its anti-inflammatory action in WAT by directly targeting the 3ʹUTR of the transcription factor signal transducer and activator of transcription 1 (STAT1), resulting in lower endogenous STAT1 mRNA, protein expression and activity. Thus, miR-30a limits the STAT1-dependent signaling pathway mediating the pro-cytokines actions. In addition, miR-103 and miR-107 were recently shown to promote endoplasmic reticulum stress-mediated apoptosis in preadipocyte by directly suppressing the expression of Wnt3a, an activator of the canonical Wnt/β-catenin pathway [52]. Therefore, these miRNAs might be involved in regulating the size of the preadipocyte population in WAT. Altogether, these recent studies clearly illustrate that in addition to perturbing signaling pathways interfering with adipogenesis, miRNAs also impact mature WAT homeostasis.

Worth mentioning, miRNAs are also detected in the plasma and could serve as important biomarkers for the diagnosis of specific diseases, although their role in the circulation is not fully understood. In this view, plasma miRNAs could serve as potential biomarkers for morbid obesity and related complications as shown by the profile of specific plasma miRNAs [53]. Indeed, increased levels of miR-142-3p, miR-140-5p, miR-222 miR-143 and miR-130, and decreased concentrations of miR-532-5p, miR-423-5p, miR-520c-3p, miR-146a, and miR-15a were strongly associated with elevated circulating adipokines and leptin levels, body mass index as well as metabolic syndrome biomarkers in individuals with morbid obesity [53]. In addition, dysregulated levels of circulating miRNAs in obesity are normalized upon acute weight loss [54], further emphasizing that miRNAs could be useful clinical biomarkers to predict the development of obesity-related complications or to assess the efficiency of promising strategies inducing weight loss.

## Long non-coding RNAs (lncRNAs)

A second class of ncRNAs, the lncRNAs, is arbitrarily defined based on their size larger than 200 nucleotides with no evidence for coding potential. LncRNAs regulate gene expression in cells and tissues as well as at various stages during development [55,56]. Like miRNAs, lncRNAs impact gene expression by binding to mRNA, however, they also bind to DNA to interfere with gene transcription. Furthermore, lncRNAs assist the formation of protein complexes involved in chromatin modification and titration of proteins away from their targets or their normal site of action. Finally, they could contribute to regulating gene expression through miRNA decoys. The biological function of most lncRNAs remains to be elucidated. Nevertheless, many lncRNAs are differentially expressed during adipogenesis and increasing evidence supports a significant role for lncRNAs in regulating this process [57,58].

The first lncRNA relevant to adipogenesis was reported in 2010 with the discovery that the non-coding RNA, steroid receptor RNA activator (SRA), behaves like a coactivator of PPARγ target genes expression by interacting with PPARγ [59]. Since this discovery, additional lncRNAs were reported to be enriched in adipose tissue and strongly induced during adipogenesis [57]. In accord, several studies identified PU.1 antisense, NEAT1, ADINR, ADNCR, H19, U90926, MIR31HG, and MEG3 [60–67] as lncRNAs that participate in multiple networks driving expression and function of genes regulating adipogenic differentiation. Furthermore, recent studies reported a role for lncRNAs ASMER and Plnc1 in adipocyte differentiation [68,69]. Indeed, these two lncRNAs were also upregulated in adipose tissue of obese mice and humans and involved in controlling the expression of genes in the adipogenic master transcriptional network, such as PPARγ.

## Conclusion

Molecular mechanisms that prevent adipogenesis may serve as powerful targets for therapeutic interventions in obesity. In this review, we present recent advances in the field of adipogenesis by highlighting newly discovered mechanisms involved in limiting adipocyte differentiation (Figure 1). Indeed, several recent studies support that differentiation of adipocyte precursor cells into adipocytes may be restricted by increasing the population of Aregs cells, promoting PDGFRα activation and signaling, and modulating expression of specific ncRNAs such as miRNAs and/or lncRNAs. In this perspective, further investigation is required to identify the anti-adipogenic factor released by Aregs cells and how it affects the differentiation of APCs. Developing cell-permeable molecules targeting intracellular negative regulators of PDGFRα signaling may also be of great interest. On the other hand, targeting APCs proliferation and differentiation by modulating the expression of specific ncRNAs, including miRNAs and lncRNAs involved in promoting or inhibiting adipogenesis may represent a powerful therapy to prevent or treat obesity. Using synthetic molecules or bioactive compounds that mimic or inhibit specific ncRNAs or modulating expression of specific ncRNAs through the powerful CRISPR-Cas9 gene editing approach may have a significant impact on obesity therapy. Considering these potential therapeutic avenues to prevent obesity, important challenges remain considering efficacy, toxicity, and specificity in targeting APCs. Nevertheless, it is of interest to consider that combination of multiple strategies targeting molecular mechanisms harnessing adipogenesis could emerge for beneficial personalized treatment of obesity and its related complications.
